# Adverse neurobehavioral changes with reduced blood and brain cholinesterase activities in mice treated with statins

**DOI:** 10.14202/vetworld.2024.82-88

**Published:** 2024-01-08

**Authors:** Rawnaq Faris Al-Shalchi, Fouad Kasim Mohammad

**Affiliations:** Department of Physiology, Biochemistry and Pharmacology, College of Veterinary Medicine, University of Mosul, Mosul, Iraq

**Keywords:** atorvastatin, head pocking, negative geotaxis, open-field activity, rosuvastatin, simvastatin, swimming endurance

## Abstract

**Background and Aim:**

Pleiotropic effects of hypolipidemic statins with behavioral outcomes have been suggested in humans and laboratory animals. There is limited information on the neurobehavioral effects of statins in mice. The aim of the present study was to examine changes in neurobehavioral performance and cholinesterase (ChE) activity in mice after high doses of three commonly used statins (atorvastatin, simvastatin, and rosuvastatin).

**Materials and Methods:**

Two hours after vehicle (control) or statin dosing at 250, 500, 750, or 1000 mg/kg orally, each mouse was subjected to 5 min open-field activity, negative geotaxis at an angle of 45°/60 s, 5 min head pocking, and forced swimming endurance. Plasma, erythrocyte, and brain ChE activities were determined spectrophotometrically 2 and 24 h after oral dosing of statins at 500 and 1000 mg/kg.

**Results:**

The statins variably, but dose-dependently and significantly (p < 0.05) delayed the latency to move in the open-field arena, decreased locomotion and rearing, reduced head pocking, and delayed negative geotaxis performance. However, statins significantly increased the duration of forced swimming and decreased the duration of immobility in the swimming tank. Statins significantly and dose-dependently decreased plasma, erythrocyte, and brain ChE activity 2 and 24 h after dosing. Plasma and brain ChE activities recovered by 5%–32.9% and 5.7%–14.4% 24 h later from the 2 h ChE values, respectively.

**Conclusion:**

High doses of statins differentially modulate neurobehavioral outcomes in mice in association with reduced plasma, erythrocyte, and brain ChE activity. Plasma or erythrocyte ChE may be used for biomonitoring of the adverse/therapeutic effects of statins.

## Introduction

Statins are a category of drugs with different pharmacokinetic, pharmacodynamic, and pleiotropic characteristics that are clinically used in humans, mainly for the treatment of hypercholesterolemia [1, 2]. They lower blood lipids by inhibiting the activity of hydroxyl-methyl-glutaryl-coenzyme A reductase, a rate-limiting enzyme in cholesterol synthesis found in the liver [1, 2]. Numerous studies have shown intriguing aspects of statins that are not related to their hypolipidemic effects, such as actions on the central nervous system and peripheral neuromuscular functions [1, 3, 4]. These actions include modulation of the central and peripheral cholinergic system [4, 5–7]. Statins have been shown to have different effects on blood or brain cholinesterase (ChE) activity [5–11]. These actions could be pertinent to the type of statin in use, dosage applied, response of the animal species versus humans, and the type of ChE in the blood or brain tissue-true versus pseudo-ChE [6, 8–12].

Interestingly, the effects of different statins on ChE activity do not appear to be consistent in both *in vitro* [5, 8] and *in vivo* [6, 8, 12] studies. The conflicting effects of statins on plasma, erythrocyte, and brain ChE activities have been reported in the form of either increase/decrease, or no effect [6–12]. The ability of statins to centrally modulate or inhibit ChE activity suggests the possibility of beneficial effects of statins in cases of dementia [4, 13].

Considering that the anti-ChE effects of statins were not consistent across the statins/tissues or species examined [6–12], another aspect of the pleiotropic effects of statins is behavioral modulation in patients and laboratory animals receiving statin therapy. Behavioral effects included aggression, depression, memory loss, and confusion [14–16]. Several experimental studies have reported neurobehavioral changes in laboratory animals, such as mice, rats, and young chicks treated with different statins [17–22]. These behavioral changes induced by statins include modulation of memory function [17], antidepressant action [18], alterations of general activity, swimming behavior, and epilepsy [20] in mice, reduction of addiction risk [19], and social behavior [22] in rats, and reduced xylazine-ketamine anesthetic duration with reductions of anti-ChE toxicity in chicks [21]. However, limited information is available on possible behavioral changes in laboratory animals with changes in blood and brain ChE activities.

Within this context, the aim of the present study was to collectively examine possible adverse neurobehavioral changes induced by high doses of three commonly used statins (atorvastatin, simvastatin, and rosuvastatin) in clinical practice [1, 2], considering changes in blood and brain ChE activities, in mice.

## Materials and Methods

### Ethical approval

This study was reviewed and approved by the Departmental Scientific Committee on Research and Animal Care and Use. The Committee of Postgraduate Studies at the College of Veterinary Medicine, University of Mosul, Iraq, approved (No. 2144, November 2, 2022) the present study according to the institutional regulations and ethics on the use of laboratory animals and their handling in biomedical research in compliance with the ARRIVE guidelines (https://www.nc3rs.org.uk/arrive-guidelines) and the guide for the care and use of laboratory Animals (https://www.ncbi.nlm.nih.gov/books/NBK54050/).

### Study period and location

The present study was conducted from Novembe-2022 to July-2023 at the Department of Physiology, Biochemistry and Pharmacology, College of Veterinary Medicine, University of Mosul, Iraq.

### Animals

Male Swiss-origin mice (age 100–120 days; weight 30–35 g) were used. The rodents were housed at temperatures of 20–24°C and a 12-h light/dark cycle, with free access to laboratory food and water. All experiments were conducted between 10 a.m. and 2 p.m.

### Drugs

Atorvastatin, simvastatin, and rosuvastatin were obtained from the state company for drugs industry and medical appliances, Samarra, Iraq. The required drug concentrations were freshly prepared using distilled water as a vehicle for oral dosing by a gavage needle at dose rates of 250, 500, 750, and 1000 mg/kg of body weight in a volume of 10 mL/kg of body weight on the experimental day. These statin doses were predetermined in preliminary experiments in mice and were selected for use in the present study because they did not induce overt signs of toxicosis; however, being sufficiently high, they allowed us to delineate neurobehavioral alterations in animal behavioral paradigms.

### Animal treatment and behavioral measurements

**Figure-1:**
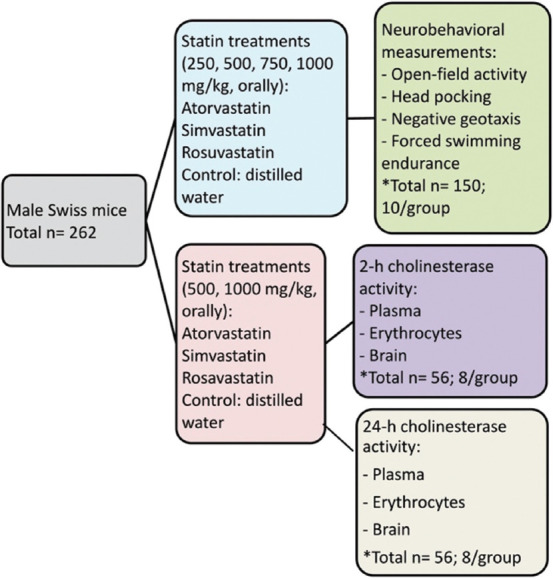
The study design, statin treatments, and neurobehavioral measurements work flow.

Mice were randomly divided into 15 groups of 10 mice/group to be orally administered atorvastatin, simvastatin, and rosuvastatin at the dose rates mentioned above (Figure-1) orally with distilled water (control). Two hours after vehicle or drug administration, each mouse was separately subjected to neurobehavioral tests that included 5 min open-field activity [23], negative geotaxis performance at an angle of 45° within 60 s [24], 5 min head pocking behavior [25], and a single-forced swimming endurance (despair) test [26].

### Determination of ChE activity

In another experiment (Figure-1), mice were randomly divided into 14 groups of 8 mice/group (2 h or 24 h) for oral dosing with distilled water (control) and treatment groups with atorvastatin, simvastatin, and rosuvastatin at dose rates of 500 mg/kg and 1000 mg/kg. Two and 24 h after the dosing of distilled water or statin, blood samples were obtained from the retro-orbital plexus under terminal ether anesthesia into heparinized capillary tubes. Blood samples were centrifuged at 900× *g* for 15 min to separate the plasma; erythrocytes were washed with physiological saline solution. The whole brain was homogenized with normal saline (1:9) at a speed of 400 rounds/s using a homogenizer (OMNI Bead Ruptor, OMNI International, USA). Cholinesterase activity in the plasma, erythrocytes, and whole brain was determined spectrophotometrically using commercial kits (Elabscience Biotechnology Inc., Houston, TX, USA).

### Statistical analysis

Data were statistically analyzed using the statistical software package Past 4.13 (https://www.nhm.uio.no/english/research/resources/past/). Parametric data consisting of multiple means were statistically analyzed using analysis of variance followed by least significant difference test. Frequency data were analyzed using Fisher’s exact probability test, and non-parametric data were analyzed using the Kruskal–Wallis test, followed by Dunn’s test. The level of statistical significance was set at p ≤ 0.05.

## Results

### Neurobehavioral effects of statins

As shown in Table-1, the three statins (atorvastatin, simvastatin, and rosuvastatin) variably but dose-dependently and significantly (p < 0.05) changed open-field activities of mice 2 h after oral dosing compared with respective control values (vehicles). Significant delays in the latency to move from the central square in the open-field arena, decreases in locomotor activity (squares crossed for 5 min), and the frequency of rearing for 5 min were observed. In line with the reduced open-field activities, statin-treated mice in a dose-dependent manner showed significant reductions in head pocking behavior and delayed negative geotaxis performance at an angle of 45°. However, statins significantly increased the duration of forced swimming and decreased the duration of immobility in the swimming tank (Table-2).

**Table-1:** Open-field activity in mice 2 h after statin treatments.

Treatment groups (mg/kg, body weight, orally)	Open-field activity		

	Latency to move (s)	Squares crossed/5 min	Rearing/5 min
Atorvastatin			
0	0.19 ± 0.03	128.4 ± 0.7	24.6 ± 0.6
250	0.30 ± 0.03	122.7 ± 1.1*	23.9 ± 0.5
500	0.52 ± 0.02*^a^	100.8 ± 0.9*^a^	13.9 ± 0.6*^a^
750	0.60 ± 0.03*^ab^	66.6 ± 2.1*^ab^	3.0 ± 0.4*^ab^
1000	0.78 ± 0.02*^abc^	42.2 ± 6.7*^abc^	1.0 ± 0.3*^abc^
Simvastatin			
0	0.13 ± 0.02	129.9 ± 1.9	20.5 ± 0.5
250	0.17 ± 0.02	125.6 ± 1.8	19.8 ± 0.6
500	0.40 ± 0.03*^a^	115.7 ± 1.3*^a^	12.7 ± 0.4*^a^
750	0.58 ± 0.03*^ab^	85.1 ± 2.2*^ab^	4.7 ± 0.4*^ab^
1000	0.68 ± 0.02*^abc^	52.3 ± 2.4*^abc^	2.1 ± 0.2*^abc^
Rosuvastatin			
0	0.14 ± 0.02	128.5 ± 1.1	24.3 ± 0.5
250	0.22 ± 0.02	125.6 ± 1.2	21.7 ± 0.6*
500	0.42 ± 0.02*^a^	112.1 ± 1.6*^a^	15.0 ± 0.9*^a^
750	0.57 ± 0.04*^ab^	87.3 ± 1.8*^ab^	5.1 ± 0.6*^ab^
1000	0.68 ± 0.03*^abc^	36.7 ± 2.04*^abc^	3.5 ± 0.3*^abc^

Values are mean ± standard error of 10 mice/group. The 0 treatment group is the control group treated with distilled water (10 mL/kg, body weight).

* Significantly different from the respective control value, p < 0.05.

a Significantly different from the respective value of the 250 mg/kg treatment group, p < 0.05. ^b^Significantly different from the respective value of the 500 mg/kg treatment group, p < 0.05. ^c^Significantly different from the respective value of the 750 mg/kg treatment group, p < 0.05

**Table-2:** Neurobehavioral outcome in mice 2 h after statin treatments.

Treatment groups (mg/kg, body weight, orally)	Head pocking/5 min	Negative geotaxis (s)	Forced swimming	

			Duration of initial swimming (min)	Duration of immobility (s)
Atorvastatin				
0	23.2 ± 0.7	2.4 ± 0.4	2.22 ± 0.05	35.5 ± 0.8
250	20.6 ± 0.7*	2.7 ± 0.6	2.36 ± 0.11	34.2 ± 1.2
500	6.8 ± 0.3*^a^	11.1 ± 0.8*^a^	3.38 ± 0.07*^a^	18.0 ± 0.6*^a^
750	4.8 ± 0.4*^a^	31.3 ± 0.8*^ab^	4.01 ± 0.08*^ab^	3.8 ± 0.5*^ab^
1000	2.1 ± 0.2*^abc^	47.4 ± 2.1*^abc^	4.46 ± 0.08*^abc^	1.6 ± 0.2*^abc^
Simvastatin				
0	24.2 ± 0.8	1.8 ± 0.2	2.34 ± 0.08	34.2 ± 0.6
250	17.8 ± 0.4	3.2 ± 0.2	2.66 ± 0.05*	34.1 ± 0.5
500	9.8 ± 0.6*^a^	29.4 ± 1.3*^a^	3.28 ± 0.90*^a^	21.5 ± 1.3*^a^
750	6.5 ± 0.4*^ab^	51.3 ± 1.2*^ab^	4.36 ± 0.80*^ab^	5.1 ± 0.5*^ab^
1000	3.1 ± 0.4*^abc^	55.5 ± 1.0*^abc^	5.25 ± 0.10*^abc^	2.2 ± 0.2*^abc^
Rosuvastatin				
0	24.0 ± 0.8	1.4 ± 0.2	2.35 ± 0.06	34.2 ± 0.6
250	21.1 ± 0.5*	4.5 ± 0.6*	2.25 ± 0.05	32.8 ± 0.5
500	10.6 ± 0.7*^a^	31.9 ± 1.4*^a^	3.13 ± 0.05*^a^	24.1 ± 1.0*^a^
750	7.0 ± 0.4*^ab^	50.8 ± 1.3*^ab^	4.34 ± 0.08*^ab^	11.1 ± 0.8*^ab^
1000	3.7 ± 0.4*^abc^	50.5 ± 0.9*^abc^	4.82 ± 0.09*^abc^	4.6 ± 0.5*^abc^

Values are mean ± standard error of 10 mice/group. The 0 treatment group is the control group treated with distilled water (10 ml/kg, body weight).

* Significantly different from the respective control value, p < 0.05.

a Significantly different from the respective value of the 250 mg/kg treatment group, p < 0.05. ^b^Significantly different from the respective value of the 500 mg/kg treatment group, p < 0.05. ^c^Significantly different from the respective value of the 750 mg/kg treatment group, p < 0.05

### Effects of statins on ChE activity

Plasma ChE activity was significantly and dose-dependently decreased 2 h post-treatment by 25%–34% and 37%–51%, respectively, compared with the respective control values in mice orally dosed with the three statins atorvastatin, simvastatin, and rosuvastatin, each at 500 and 1000 mg/kg, respectively (Table-3). Twenty-four hours after statin dosing, plasma ChE activity decreased by 19%–30% and 24%–34%, respectively, compared with the respective control value (Table-3). When erythrocyte ChE activity was determined in statin-treated mice, the 2 h decrease in activity was significant by 11%–12% and 24%–25%, respectively, and the 24 h reduction values were 14%–16% and 25%–30%, respectively, relative to the respective control values (Table-4). Similarly, the 2 h brain ChE activity was significantly reduced in statin-treated mice by 13%–17% and 27%–31%, respectively, and that of the 24 h after treatment was 8% for the 500 mg/kg dose groups, and 22% to 24% in the 1000 mg/kg dose groups, when compared with the respective control value (Table-5). Twenty-four hours after statin treatments, plasma and brain ChE activities recovered from the 2 h values to gain more activity by 5%–32.9% and 5.7%–14.4%, respectively (Tables-3 and 5). Erythrocytes did not recover from the depressed values when determined 24 h later (Table-4).

**Table-3:** Plasma cholinesterase activity (U/mL) in mice dosed orally with statins.

Treatment group (mg/kg)	2 h	24 h	% recovery from the 2 h values
Distilled water	54.506 ± 1.432	52.961 ± 0.941	0
Atorvastatin (500)	40.972 ± 0.344*	43.035 ± 0.435*^†^	5.0
Simvastatin (500)	38.802 ± 0.567*	36.952 ± 1.422*^a^	0
Rosuvastatin (500)	35.976 ± 0.518*^a^	39.778 ± 0.710*^†ab^	10.6
Atorvastatin (1000)	30.430 ± 0.569*^abc^	40.430 ± 0.491*^†ab^	32.9
Simvastatin (1000)	34.127 ± 0.866*^abd^	38.693 ± 0.614*^†a^	13.4
Rosuvastatin (1000)	26.515 ± 0.958*^abcde^	34.780 ± 0.677*^†acde^	31.2

Values are mean ± standard error of 8 mice/group.

* Significantly different from the respective control group, p < 0.05.

† Significantly different from the respective 2 h cholinesterase activity, p < 0.05.

a Significantly different from the respective atorvastatin 500 mg/kg dose group, p < 0.05. ^b^Significantly different from the respective simvastatin 500 mg/kg dose group, p < 0.05. ^c^Significantly different from the respective rosuvastatin 500 mg/kg dose group, p < 0.05. ^d^Significantly different from the respective atorvastatin 1000 mg/kg dose group, p < 0.05. ^e^Significantly different from the respective simvastatin 1000 mg/kg dose group, p < 0.05

**Table-4:** Erythrocyte cholinesterase activity (U/mL) in mice dosed orally with statins.

Treatment group (mg/kg)	2 h	24 h
Distilled water	77.835 ± 0.888	76.222 ± 0.941
Atorvastatin (500)	69.060 ± 0.509*	65.468 ± 0.790*^†^
Simvastatin (500)	68.615 ± 0.776*	64.471 ± 0.697*^†^
Rosuvastatin (500)	68.589 ± 0.889*	63.936 ± 0.951*^†^
Atorvastatin (1000)	58.725 ± 0.561*^abc^	56.992 ± 0.674*^abc^
Simvastatin (1000)	57.878 ± 0.785*^abc^	53.518 ± 0.689*^†abc^
Rosuvastatin (1000)	59.127 ± 0.647*^abc^	53.215 ± 0.745*^†abcd^

Values are mean ± standard error of 8 mice/group.

* Significantly different from the respective control group, p < 0.05.

† Significantly different from the respective 2 h cholinesterase activity, p < 0.05. ^a^Significantly different from the respective atorvastatin 500 mg/kg dose group, p < 0.05. ^b^Significantly different from the respective simvastatin 500 mg/kg dose group, p < 0.05. ^c^Significantly different from the respective rosuvastatin 500 mg/kg dose group, p < 0.05. ^d^Significantly different from the respective atorvastatin 1000 mg/kg dose group, p < 0.05

**Table-5:** Brain cholinesterase activity (U/mg protein) in mice dosed orally with statins.

Treatment group (mg/kg)	2 h	24 h	% recovery from the 2 h values
Distilled water	75.418 ± 0.958	75.672 ± 0.712	0.3
Atorvastatin (500)	65.797 ± 0.964*	69.887 ± 0.628*	6.2
Simvastatin (500)	62.671 ± 0.651*^a^	69.812 ± 0.619*^†^	11.4
Rosuvastatin (500)	64.020 ± 0.767*	69.453 ± 0.593*^†^	8.5
Atorvastatin (1000)	55.277 ± 0.706*^abc^	58.725 ± 0.561*^†abc^	6.2
Simvastatin (1000)	54.777 ± 0.879*^abc^	57.878 ± 0.785*^†abc^	5.7
Rosuvastatin (1000)	51.668 ± 0.379*^abcd^	59.127 ± 0.647*^†abc^	14.4

Values are mean ± standard error of 8 mice/group.

* Significantly different from the respective control group, P < 0.05.

† Significantly different from the respective 2 h cholinesterase activity, p < 0.05.

a Significantly different from the respective atorvastatin 500 mg/kg dose group, p < 0.05. ^b^Significantly different from the respective simvastatin 500 mg/kg dose group, p < 0.05. ^c^Significantly different from the respective rosuvastatin 500 mg/kg dose group, p < 0.05. ^d^Significantly different from the respective atorvastatin 1000 mg/kg dose group, p < 0.05

## Discussion

The main finding of the present study is that atorvastatin, simvastatin, and rosuvastatin cause general acute neurobehavioral changes after 2 h of dosing. These effects could be an additional pleiotropic outcome of statins appended to the already known adverse behavioral changes not related to hypolipidemic actions [1, 3, 20]. The reported adverse behavioral effects of statins in humans include episodes of aggression, depression, loss of memory, or confusion [14–16], and in laboratory animals, they have been reported to cause memory alteration, changes in locomotor, social, and swimming activities, modulation of drug addiction, and antidepressant effects [17–22]. In the present study, statins reduced general locomotor activity (open-field activity and negative geotaxis performance), possible anxiolytic effect (head pocking behavior), and antidepressant action (swimming endurance). These neurobehavioral changes collectively reported in the present study were dose-dependent and suggest a multifunctional aspect of statin-induced behavioral modulation in mice, in support of previous reports in laboratory animals [17–22]. Furthermore, the present behavioral results, based on head pocking and swimming endurance as well as the current evidence in the literature [18, 20–22], suggest that the depressive effects of statins on locomotor activity vary depending on their potential antidepressant properties. Further, preclinical and possible therapeutic exploration of this concept in animal models should be explored.

The doses of the three statins used in the present study were higher than those clinically used in humans [1–4]. However, the choice of statin doses in the present study was based on preliminary experiments in mice that did not show obvious signs of toxicosis. Emerging data indicate that statins are well tolerated even at high doses [27], which is an added advantage for any animal model that can tolerate high doses of statins to facilitate behavioral changes [9, 21]. In addition, species variation is expected in experimental animals in response to statin dosages, and they differ in pharmacological and toxicological profiles, especially when the end point effect is not the plasma cholesterol level; statin doses could be up to 10–80 times larger than those of humans [8, 9, 11, 17, 21, 28].

Reduced ChE activity in the plasma, erythrocytes, and brain represents another important finding of the present study in mice orally administered single doses of each of the three statins (500 and 100 mg/kg). These results are consistent with the previous studies on ChE activity in humans [10], rodents [6, 7, 9, 11], and chicks [8] treated with single or repeated doses of statins. In contrast to the present study, we observed selective and differential effects of statins on ChE activity in the studies mentioned above. In the present study, high doses of statins were administered, and the results suggest generalized depressive effects of statins in conjunction with behavioral alterations on ChE activities in the plasma, erythrocytes, and brain. These results further support previous findings on reduced ChE activity as a result of statin therapy and highlight the fact that high doses of statins may modulate the enzyme activity toward depression, which remains to be determined. Most statins reduce ChE activity; however, other statins may have no effect or even increase ChE activity [6–12].

The three tissue ChE activities (plasma, erythrocytes, and brain) examined in the present study represent pseudo-ChE in plasma (some in the brain) and true ChE in erythrocytes and brain [29, 30]. They are target subjects to various extents of pesticide inhibition [29, 30]. They also recover from inhibition in a differential manner, depending on the type of inhibitor and the enzyme source, with fast recovery observed in plasma ChE [30]. Although it is too early to draw a cause-and-effect relationship from the present findings, several reports have indicated that inhibited brain ChE activity is associated with neurobehavioral changes such as increased anhedonia (preference to sucrose), depressed open-field activity, learning and memory impairment, and forced swimming behavioral changes in rodents [31]. The pharmacokinetic, pharmacodynamic, and pleiotropic profiles of statins differ [1, 2]. Extensive *in vitro* and *in vivo* assessments of ChE activity in association with neurobehavioral outcomes are warranted to understand the neuronal actions of statins, taking into account their potential neurotoxicity versus beneficial effects in cases of dementia [3, 4, 13–15].

## Conclusion

Our results suggest that high-dose statins differentially modulate neurobehavioral outcomes in mice in association with reduced ChE activity in the brain. Changes in ChE activity in plasma and erythrocytes indicate the potential for biomonitoring of statin adverse/therapeutic effects.

## Authors’ Contributions

RFA: Performed animal experiments and laboratory assays; conducted a literature search, performed statistical analyses, and shared in drafting the manuscript. FKM: Conceptualized and designed the project, supervised the study, participated in the literature search, performed statistical analyses, and drafted the manuscript. Both authors have read, reviewed, and approved the final version of the manuscript.
